# Visualizing Influenza A Virus Infection and Host Processes Using Fluorescence Microscopy

**DOI:** 10.3390/v18101074

**Published:** 2026-09-29

**Authors:** Bernhard A. Kramer, Janin Schokolowski, Huib H. Rabouw

**Affiliations:** Oncode Institute, Hubrecht Institute-KNAW and University Medical Center Utrecht, Uppsalalaan 8, 3584 CT Utrecht, The Netherlands; b.kramer@hubrecht.eu (B.A.K.); j.schokolowski@hubrecht.eu (J.S.)

**Keywords:** fluorescence imaging, influenza A virus, live-cell imaging, viral life cycle, virus–host interaction, antiviral responses, host cell remodeling, single-cell imaging, infection heterogeneity, infection dynamics

## Abstract

Virus infections unfold through spatially organized events whose timing and progression vary among individual cells. Imaging complements population-based approaches by preserving spatial context and, in live experiments, temporal progression at single-cell and tissue scales. This review examines how imaging has advanced our understanding of virus infection, using influenza A virus (IAV) as the principal example. The review is organized around three complementary perspectives: visualization of viral components throughout infection, analysis of virus-induced host-cell and tissue remodeling, and investigation of heterogeneous antiviral responses. Together, these perspectives show how imaging preserves the spatial, temporal, and cell-to-cell context needed to relate viral processes to host-cell responses across scales. Because imaging approaches differ in what they directly measure, this review considers their complementary strengths and limitations and distinguishes descriptive observations from mechanistic conclusions.

## 1. Introduction

Virus infection is a spatially and temporally organized process in which viral life-cycle progression, host-cell remodeling, and antiviral responses continuously interact to determine infection outcome [1,2,3]. Influenza A virus (IAV) provides a clear example of this spatial and temporal organization: its life cycle requires coordinated use of distinct cellular compartments, such as the endosomal compartment, the nucleus, intracellular trafficking networks, and the plasma membrane [4,5]. Importantly, infection progression and outcome are not uniform across infected cells. Even within genetically identical cell populations, individual cells can follow distinct trajectories due to differences in incoming virus particles, viral gene expression dynamics, host-cell state, and antiviral response activation [2,6,7,8]. As a result, measurements that average across populations can obscure cell-to-cell heterogeneity, including divergent infection trajectories and rare infection states.

Recent advances in single-particle tracking, live-cell microscopy, multiplexed imaging of viral RNAs, super-resolution microscopy, and tissue-scale imaging have begun to fill important gaps between population-averaged descriptions of infection and the spatially and temporally organized events occurring in individual cells [9,10,11]. These imaging modalities reveal when and where viral and host processes occur, resolve heterogeneous infection trajectories, and allow multiple features of infection to be related within the same cell or tissue. However, insights obtained through imaging remain distributed across studies focused on different viruses, life-cycle stages, host processes, imaging modalities, and spatial scales. Here, we integrate diverse imaging studies to assess how fluorescence imaging has collectively expanded our understanding of virus infection, drawing on electron microscopy, tomography, and correlative approaches where they complement or extend fluorescence-based findings. Throughout, imaging refers to point-scanning or spinning-disk confocal fluorescence microscopy unless otherwise specified.

With a primary focus on IAV, this review draws on selected examples from other viruses that offer relevant methodological or biological insights and examines infection from three complementary perspectives: viral components and infection progression, virus-induced remodeling of host-cell organization, and antiviral responses. Together, these perspectives show how viral life-cycle progression, host-cell remodeling, and antiviral responses are interconnected and shape infection outcomes (Figure 1).

## 2. Imaging Viral Components

### 2.1. Visualizing Virion Attachment, Trafficking, and Membrane Fusion

IAV infection begins with attachment to susceptible cells. To establish productive attachment, virions must navigate extracellular environments while avoiding repeated engagement with decoy receptors [15,16,17]. Hemagglutinin (HA) binds terminal sialic acid residues on cellular and extracellular glycoconjugates, whereas neuraminidase (NA) cleaves these residues; accordingly, perturbation of either HA receptor binding or NA enzymatic activity reduces virion infectivity [18,19]. However, extracellular navigation is a spatial process, and endpoint measurements could not reveal how HA and NA together direct virion movement. Labeling virions with lipophilic membrane dyes enabled tracking of individual particles across sialylated surfaces and through complex extracellular environments (Figure 2A) [15,20]. Tracking single virions revealed that coordinated receptor binding by HA and receptor cleavage by NA promote persistent movement through sialylated environments by preventing repeated attachment to previously encountered receptors [15], likely facilitating successful engagement with susceptible cells. Following productive attachment, IAV particles are internalized by endocytosis, and subsequent acidification of endosomal compartments triggers fusion between viral and host membranes. Fluorescent labeling allowed visualization of virion attachment to the host cell plasma membrane and transport through the endosomal network, while self-quenching lipid dyes provided a direct readout of the timing and location of membrane fusion [21,22,23,24]. Combining single-virion tracking with detection of membrane fusion demonstrated that fusion does not occur immediately after uptake. Instead, virions undergo microtubule-dependent transport through the endosomal network toward the perinuclear region before endosomal acidification triggers membrane fusion [21]. Thus, single-virion tracking and membrane-fusion measurements established that successful entry depends on completion of multiple trafficking and maturation steps before genome release.

### 2.2. Following Incoming Viral Ribonucleoproteins (vRNPs) from Fusion to Nuclear Import

The events linking fusion to nuclear delivery have remained difficult to study because incoming vRNPs are present in low copy numbers. Early immunofluorescence (IF) studies of vRNPs performed at a high multiplicity of infection (MOI) suggested that nuclear import is efficient and rapid [25]. Subsequent application of single-molecule fluorescence in situ hybridization (smFISH) enabled visualization of viral RNA segments during the earliest stages of infection, revealing vRNP subcellular localization, colocalization, and nuclear import [26]. However, because these experiments required high-MOI infections and relied on fixed-cell snapshots, neither approach could track individual incoming vRNPs or reveal the timing of the intermediate steps linking membrane fusion to nuclear delivery. An important advance came with quantum-dot labeling of the polymerase acidic protein (PA) subunit of the viral polymerase, allowing the first direct visualization of individual incoming vRNPs in living cells [27]. Quantum-dot tracking of individual incoming vRNPs indicated that membrane fusion occurs predominantly near the nucleus, where individual vRNPs are released from the incoming bundle before rapidly being imported into the nucleus. Recently, live vRNP labeling using fluorescent nanobodies targeting the nucleoprotein (NP) within vRNP complexes (VISUN; vRNP imaging of unmodified negative-strand viruses) enabled visualization of all incoming vRNPs. While VISUN does not require viral engineering or in-virion labeling procedures, experiments have to be conducted in cell lines or organoids expressing the fluorescently labeled anti-NP nanobody intracellularly (Figure 2B) [12]. VISUN confirmed rapid and complete post-fusion vRNP debundling and demonstrated that the majority of incoming vRNPs reach the nucleus within minutes of membrane fusion. Because VISUN detects vRNPs only after membrane fusion, it does not capture pre-fusion endosome trafficking, and the spatial distribution of fusion events has so far been addressed only by quantum-dot tracking. Taken together, the data on IAV entry suggest that the transition from membrane fusion to nuclear delivery does not pose a major barrier to productive infection.

### 2.3. Visualizing Viral Protein Expression and Localization

Viral gene expression results in the accumulation of viral proteins, which can be visualized by IF and used as markers of infection progression. Beyond measuring protein abundance, fluorescence imaging revealed that many influenza proteins undergo characteristic changes in subcellular localization that reflect their changing functions throughout infection [28,29]. For example, observation of nuclear accumulation of the matrix protein 1 (M1) helped define its role in terminating viral replication and promoting nuclear export of vRNPs [30]. Following completion of genome replication, NP redistributes from the nucleus to the cytoplasm during vRNP nuclear export. Imaging of NP localization thus directly visualizes the transition from viral genome replication to cytoplasmic transport [31]. Taken together, viral infection is not a static process of protein production, but involves coordinated spatial redistribution of viral factors between cellular compartments as infection progresses. Live-cell imaging complements fixed-cell approaches by resolving the temporal dynamics of viral protein expression and localization. Approaches for tracking protein expression or localization in living cells broadly fall into two categories: viruses that express directly tagged viral proteins, and reporter viruses that express fluorescent or luminescent proteins as indirect readouts of viral gene expression (Figure 2C).

Direct visualization of IAV proteins was achieved using recombinant IAVs expressing genetically tagged polymerase basic protein 2 (PB2) [32,33], PA [34], nonstructural protein 1 (NS1) [35,36], or structural proteins [37]. Because many RNA viruses, including IAV, tolerate only limited genetic perturbation, particularly large insertions encoding fluorescent proteins, recombinant fluorescent viruses require extensive validation and optimization to ensure that tagging does not compromise viral fitness or genetic stability [33]. Live tracking of fluorescently labeled polymerase-containing vRNPs revealed directed, microtubule-dependent transport through the cytoplasm following nuclear export (Figure 2E) [32,33,34]. Real-time visualization of viral structural proteins enabled direct observation of virion assembly and release at the plasma membrane [37]. Because fluorescence becomes detectable only after sufficient accumulation of the tagged protein, direct protein-labeling approaches are best suited for studying later stages of infection and are generally less informative for the earliest stages.

Reporter viruses expressing fluorescent or bioluminescent proteins can be used to monitor infection spread continuously in cultured cells and animal models, revealing how viral dissemination depends on viral genotype, host antiviral responses, and tissue context [35,38,39,40,41]. At the single-cell level, substantial variation in the onset and rate of viral gene expression has been shown among individual infected cells despite synchronous infection [39]. Multicolor reporter viruses enabled coinfection to be visualized within individual cells and functional interactions between coinfecting viruses to be monitored [42]. Interactions between coinfecting viruses can either inhibit or promote productive infection. For example, live imaging showed that primary infection progressively reduces susceptibility to secondary infection through superinfection exclusion [42]. Conversely, coinfecting viruses can complement incomplete viral genomes by supplying missing functions in trans, thereby supporting productive infection [43].

### 2.4. Imaging Viral RNA Synthesis and Genome Replication

Although viral protein expression reports on infection progression, it provides only an indirect view of the underlying transcriptional and replication program. IAV infection progression is governed by the synthesis of distinct viral RNA species, including viral mRNAs, full-length complementary RNAs (cRNAs) that serve as replication templates, and negative-sense genomic RNAs (vRNAs). Biochemical approaches established the average temporal order of viral transcription and genome replication [44,45,46,47,48]. RNA imaging introduced a complementary perspective by allowing viral RNAs to be detected directly in individual infected cells while retaining information about their intracellular localization (Figure 2D). Initial RNA FISH studies examined the spatial organization of the eight viral genome segments within virions and showed that individual virions generally package one copy of each genome segment [49]. Subsequent multiplexed RNA imaging addressed the dynamics of viral transcription and replication. For example, multiplexed error-robust FISH (merFISH) was used to simultaneously quantify all eight viral mRNAs in individual infected cells, revealing segment-specific differences in cytoplasmic mRNA abundance due to both differential transcription and nuclear export [50].

More recently, in situ sequencing with multiple direct RNA-assisted padlock probing (mudRapp-seq) enabled simultaneous visualization of viral mRNAs and genomic RNAs, allowing transcriptional output and genome replication to be quantified on a segment-by-segment basis [51]. The mudRapp-seq measurements revealed pronounced segment-specific differences in transcriptional output despite largely similar genome replication dynamics. Direct live imaging of viral RNA dynamics and trafficking remains technically challenging. Current live RNA imaging strategies (e.g., PP7/MS2 stem-loop systems, RNA aptamers, or CRISPR-dCas13 imaging) rely on large engineered RNA insertions or exogenous probe delivery [52,53] and are difficult to implement in IAV because of its compact genome. Insertions should not affect coding sequences, splice sites, or packaging signals for particle assembly. Because packaging signals in particular remain incompletely mapped, the insertion of bulky imaging tags risks disrupting the NP-coated RNA architecture that may lead to segment assembly defects and subsequent impaired viral fitness [5,54]. Improved mapping of packaging signals would allow insertion sites to be chosen rationally, while smaller imaging modules would reduce the risk of disrupting vRNP architecture. Whether these advances would be sufficient to enable segment-specific live vRNA imaging, or whether other constraints dominate, remains unclear.

### 2.5. Continuous Live Imaging of vRNPs

IAV genome segments can instead be visualized indirectly, by targeting the associated proteins within the vRNP complex. Earlier protein-labeling strategies enabled imaging of either incoming [27] or newly synthesized [32,33,34] vRNPs, but not continuous tracking throughout infection. More recently, imaging of cells expressing fluorescent nanobodies targeting NP (VISUN) enabled continuous visualization of vRNPs throughout infection and direct measurement of the timing of genome replication and nuclear export in living cells (Figure 2B) [12]. Because individual infection trajectories can be followed from entry onward, VISUN also makes it possible to identify and quantify abortive infections, defined as infections that stall at a defined stage rather than completing the viral life cycle, providing a more complete picture of infection outcomes within a cell population. Finally, because VISUN does not require viral engineering, it enables direct quantitative comparison of the life cycles of genetically diverse influenza strains and clinical isolates. However, VISUN does not distinguish individual genome segments or RNA species. This tradeoff reflects the fundamental difference between VISUN and fixed-cell RNA imaging approaches such as mudRapp-seq (see Section 2.4): VISUN provides continuous live-cell tracking of vRNP dynamics but cannot distinguish individual genome segments or RNA species, whereas fixed-cell approaches such as mudRapp-seq (see Section 2.4) provide strand- and segment-specific resolution but only as snapshots.

### 2.6. Imaging Genome Assembly and Progeny Production

Following nuclear export, newly synthesized vRNPs are transported toward the plasma membrane, where progeny virions are assembled. However, how assembly of the eight distinct genome segments is coordinated during transport to budding sites remained unclear. Segment-specific RNA imaging demonstrated that individual genome segments progressively colocalize during cytoplasmic transport, while live-cell imaging of fluorescently labeled polymerase-containing complexes revealed organized, directional trafficking of vRNPs along microtubules. Together, progressive segment colocalization and organized, directional vRNP trafficking support a model in which genome assembly occurs through sequential interactions on membranous compartments prior to budding rather than exclusively at the plasma membrane [26,34]. Recently, the addition of fluorescent nanobodies targeting HA to the extracellular environment has provided the first live readout of progeny virion budding (Figure 2F) [12]. Unlike the intracellular anti-NP nanobody described above, this HA nanobody is applied to the extracellular medium and requires no genetic modification of the virus or host cells. However, the HA nanobody neutralizes virions, restricting its use to single-cycle infections. Combining HA-nanobody-based detection of progeny virion budding with imaging of vRNP nuclear export demonstrated that progeny virion production follows vRNP nuclear export with minimal time delay. However, not all cells with observed vRNP nuclear export subsequently produce detectable progeny virions, revealing that budding is not a guaranteed next step after vRNP export and instead is an additional source of infection heterogeneity.

## 3. Imaging Virus-Induced Host-Cell Remodeling

Following viral processes through a host cell identifies where successive stages of infection occur but does not necessarily reveal how infection changes the cellular environment in which those stages proceed. Viral infection remodels host cells by redistributing molecular components, reorganizing organelles, constructing new reaction environments, and altering cellular and tissue architecture. Because many virus-induced cellular changes are defined by their spatial organization, temporal dynamics, or ultrastructure rather than molecular abundance alone, imaging is uniquely suited to study virus-induced host-cell remodeling. Different imaging modalities provide complementary views: fluorescence microscopy visualizes the distribution of selected molecules, live-cell imaging resolves their dynamics, electron microscopy reveals membrane architecture and ultrastructure, and tissue imaging relates cellular phenotypes to higher-order organization. Accordingly, conclusions about host-cell remodeling should reflect the specific cellular property that each imaging approach directly measures.

### 3.1. Visualizing Spatial Remodeling of Gene Expression

Cellular gene expression depends not only on molecular abundance, but also on localization of RNAs and RNA processing machinery. Both IF and smFISH can determine whether selected proteins or RNAs remain dispersed or accumulate in compartments at the single-cell level, and have been particularly useful for studying IAV-induced host shutoff, a process characterized by extensive spatial reorganization of the cellular gene-expression machinery. Combined smFISH and IF microscopy revealed depletion of cytoplasmic poly(A) RNA and nuclear accumulation of poly(A) RNA and poly(A)-binding protein 1 (PABPC1), while confocal fluorescence microscopy further showed dispersal of nuclear speckles, nuclear compartments involved in pre-mRNA processing [55,56,57]. Comparisons of cells infected with recombinant IAVs lacking expression of either NS1 or PA-X distinguished the individual spatial changes that together comprise host shutoff. Viral protein PA-X accounts for much of the cytoplasmic RNA loss, and NS1 compromises NXF1-dependent mRNA export. Consistently, nuclear PABPC1 accumulation depends on concerted PA-X and NS1 activity. Thus, combining imaging of host-shutoff-associated spatial changes with the use of engineered viruses that fail to express individual viral proteins reveals mechanistically distinct host-shutoff processes.

Although fixed-cell IF and smFISH reveal spatial reorganization of RNAs and RNA-processing machinery during infection, they do not report on RNA integrity, translation, or movement [58]. By combining live-cell imaging of viral RNA translation and replication dynamics with fixed-cell measurements of host protein synthesis, a study of coxsackievirus B3-infected cells showed that global host protein synthesis was already substantially reduced before replication of the incoming viral RNA, whereas viral translation efficiency remained stable throughout infection [59]. This illustrates how combining measurements of viral and host RNA dynamics with protein synthesis can provide functional information that complements mRNA localization measurements. Applied to influenza, similar approaches could directly link virus-induced changes in RNA localization to their consequences for protein synthesis.

### 3.2. Detection of Virus-Built Reaction Environments

IAV replication takes place in the nucleus and does not require formation of the membrane-associated replication organelles (ROs) characteristic of many cytoplasmic RNA viruses [5]. In contrast, diverse viruses such as flaviviruses, enteroviruses, and coronaviruses construct de novo membranous compartments dedicated to viral replication [60]. Imaging approaches developed to understand RO biogenesis have clarified their molecular composition, assembly dynamics, and membrane architecture. Fluorescence microscopy identified enriched viral and host components [60]. Live fluorescence imaging revealed the dynamics of RO formation [61], whereas electron tomography revealed highly organized, topologically complex membrane architecture [62]. Together, these observations showed how viruses construct and organize specialized replication environments. However, molecular enrichment or morphology do not by themselves establish organelle function, which requires complementary functional evidence.

### 3.3. Visualizing Organelle Function and Membrane Topology

Infection also remodels pre-existing organelles, creating an important interpretive distinction between changes in morphology and changes in function. Mitochondria illustrate this distinction, since they may fragment, elongate, or redistribute during infection. Yet, these morphological changes do not by themselves establish altered mitochondrial function. Specialized live reporters and fluorescence biosensors can measure membrane potential or local biochemical activity that are not apparent from mitochondrial shape or position. Live-cell imaging using mitochondrial functional reporters in IAV-infected cells linked expression of selected PB1-F2 variants to loss of membrane potential and organelle injury, but the magnitude and direction of IAV-associated mitochondrial changes depend on viral strain and experimental context [63,64,65].

Membrane topology presents a related but distinct imaging challenge: the distribution of labeled proteins does not define the membrane structures that contain them. During virion budding, for example, viral proteins accumulate at defined sites on the plasma membrane, but their localization alone does not reveal how the membrane is reshaped. Electron microscopy and tomography therefore complement fluorescence imaging by resolving distinct membrane architectures during IAV budding, including glycoprotein-associated membrane curvature, M1-dependent formation of filamentous virions, and matrix-2 protein enrichment at budding necks associated with membrane scission [66,67,68]. Together, fluorescence microscopy, electron microscopy, and tomography combine molecular localization with direct visualization of membrane architecture to reveal membrane-remodeling events that cannot be inferred from protein localization alone.

### 3.4. Following Endomembrane Trafficking

To transport progeny vRNPs from the nucleus to the plasma membrane, IAV remodels the pre-existing Rab11-dependent recycling endosome network. This remodeling of the recycling endosomal compartment was characterized by studies using several complementary imaging approaches. Fixed-cell fluorescence showed that the normally dispersed Rab11 recycling endosomal network becomes enlarged and clustered during infection [69]. Using a vRNP reconstitution system, dual-color live imaging first identified vRNP cotrafficking with Rab11-positive membranes [70]. The thin optical sectioning and reduced out-of-focus illumination provided by light-sheet microscopy enabled long-term, high-temporal-resolution volumetric imaging of Rab11-vRNA transport while minimizing photobleaching and phototoxicity, revealing that vRNP-bearing carriers move more slowly, travel shorter distances, and arrest more frequently [13]. Correlative light-electron microscopy localized vRNPs to infection-remodeled Rab11 carriers, directly connecting Rab11 redistribution with vRNP trafficking [71]. Live imaging showed that vRNP-containing inclusions deform, fuse, split, and exchange material (Figure 2E), while fluorescence recovery measurements supported rapid internal turnover [72]. The observed dynamics and rapid internal turnover of vRNP-containing inclusions are consistent with liquid-like behavior, including round morphology, coalescence on contact, and fluorescence recovery after photobleaching. However, the same readouts are expected for Rab11 endosomes held together by low-valency spatially clustered binding sites. The presence of host membranes within these inclusions adds to this ambiguity, since membranes can independently produce similar morphology and exchange behavior without requiring liquid-phase separation. Definitive assignment of the material state therefore requires complementary evidence, such as pharmacological modulation of material properties or depletion of host factors [73].

As IAV reorganizes the Rab11 compartment, Rab11-dependent cargo recycling declines. Functional assays using fluorescent transferrin as recycling cargo demonstrated that Rab11-dependent cargo transport is attenuated during IAV infection. Fluorescence imaging of Rab11 redistribution, combined with functional recycling and binding measurements, supported a model in which vRNPs compete with Rab11-interacting proteins for Rab11 binding, thereby reducing cellular recycling and contributing to Rab11 redistribution [69]. A subsequent study combining fluorescence imaging with perturbational evidence showed that ATG9A promotes the release of vRNP-bearing Rab11 endosomes from microtubules and their organization into viral inclusions, indicating a regulated reorganization mechanism not fully explained by competition for Rab11 binding alone [74]. Correlative light-electron microscopy further showed that altered Rab11 carriers associate with remodeled endoplasmic reticulum, suggesting that IAV reorganizes a broader membrane network than previously appreciated [71]. The combined evidence therefore supports regulated remodeling of the recycling endosome compartment that extends beyond Rab11-binding competition alone. Overall, IAV redirects a constitutive recycling system into a slower, spatially concentrated transport environment that supports vRNP delivery while compromising host cargo recycling. This model was established by integrating spatial, dynamic, ultrastructural, and perturbational measurements rather than any single imaging approach.

### 3.5. Imaging of Intercellular and Tissue-Scale Remodeling

Virus infection remodels host tissues at multiple spatial scales, ranging from interactions between neighboring cells to changes in tissue architecture and function. Imaging has been essential for linking intercellular and tissue-level changes to virus dissemination and pathogenesis. Confocal and stimulated emission depletion (STED) super-resolution imaging localized Rab11-associated vRNPs within actin-rich intercellular connections between infected and neighboring cells, demonstrating directed movement of viral material from infected cells toward neighboring cells [75]. Infectivity measurements confirmed that transfer through these structures seeds productive infection, identifying a potential route of cell-to-cell spread that complements extracellular virus release.

Beyond intercellular communication, imaging revealed how infection alters tissue function. Live imaging of IAV-infected airway systems demonstrated infection-associated changes in ciliary activity [76]. Longitudinal imaging of SARS-CoV-2-infected airways linked mucociliary movement to the spatial pattern of viral spread, showing that tissue-level transport processes shape infection dissemination across the airway epithelium [77]. Equivalent longitudinal imaging of IAV-infected airways could test whether mucociliary transport similarly constrains IAV spread, which is relevant given that mucus is a known barrier to influenza infection. Infection-induced remodeling also changes tissue composition. For example, the ability of light-sheet microscopy to rapidly image large, intact 3D volumes at cellular resolution enabled quantification of cell-type composition across the entire 3D architecture of Zika virus-infected cerebral organoids, revealing the loss and spatial redistribution of specific cell populations throughout the tissue [78]. Similarly, imaging showed that IAV infection depletes ciliated cells and reduces airway epithelial thickness, thereby changing the cell-type composition of the airway epithelium [79]. Yet electrical measurements indicated preserved barrier function, demonstrating that tissue composition and function can be remodeled independently during infection. These tissue-scale approaches trade molecular resolution for spatial coverage. Imaging large volumes or extended time courses limits the sensitivity needed to detect individual molecules, so the intracellular events underlying tissue-level changes generally require separate, higher-resolution measurements (see also Table 1). By linking infection to changes in intercellular communication, tissue organization, and function, imaging extends virology research beyond individual cells to higher-order processes that influence viral spread and disease progression.

## 4. Heterogeneity of Antiviral Responses

Virus-induced host-cell and tissue remodeling occurs alongside antiviral responses that can redirect infection trajectories. Cells detect virus infection through pattern-recognition receptors activated by pathogen-associated molecular patterns (PAMPs), including viral nucleic acids with aberrant structures or subcellular localization. PAMP recognition triggers antiviral responses, most notably interferon (IFN) production and the expression of hundreds of interferon-stimulated genes (ISGs) [80], while viruses counteract PAMP-induced antiviral responses by concealing viral PAMPs, sequestering replication intermediates, and disrupting antiviral signaling pathways [81]. At the single-cell level, the balance between antiviral signaling and viral evasion produces heterogeneous response activation even under uniform infection conditions [2,6], reflecting both virus-intrinsic differences in genome integrity, gene expression, or particle composition and host-cell differences in the abundance and activity of sensing components [6,82,83]. Cell-to-cell heterogeneity in antiviral responses is evident during IAV infection. IAV evades antiviral responses in most infected cells [6,84], and the PAMPs that trigger immunity remain incompletely defined. Full-length vRNPs, defective genomes, and aberrant replication products have all been implicated as potential triggers of innate immune activation [85,86,87]. Imaging provides three complementary perspectives on cell-to-cell heterogeneity in antiviral response activation. First, time-resolved imaging that follows infection progression and host responses in individual cells identifies which viral life-cycle trajectories are associated with antiviral response activation [14]. Second, imaging the spatial organization of viral components and host factors reveals how antiviral signaling is initiated, amplified, or disrupted [88]. Third, localizing the effects of interferon-stimulated restriction factors identifies the viral life-cycle stages at which antiviral responses inhibit infection [89].

### 4.1. Relating Infection Trajectories to Antiviral Response Activation

Because population-level measurements average across infected cells, they can obscure antiviral responses confined to a responding minority. Imaging at single-cell resolution can identify responding cells and relate antiviral response activation to infection progression within the same cell. By preserving spatial context, imaging can additionally relate antiviral responses in infected cells to responses in neighboring bystander cells. For example, live-cell tracking of IAV replication by VISUN, combined with fixed-cell analysis of antiviral response activation, showed that IFN-λ induction occurred predominantly in cells lacking functional NS1 expression while exhibiting high levels of vRNP replication [12]. Antiviral activation is also dependent on the nature of the viral RNA produced, since defective viral genomes arising through replication errors are associated with enhanced antiviral signaling [90]. Antiviral responses therefore do not simply correlate with the extent of viral replication, but likely depend on the balance between accumulation of immunostimulatory viral RNA and establishment of viral antagonism. Live-cell imaging allows these determinants to be separated by linking viral protein expression and infection progression, including abortive infections that stall before completing the infection cycle (see Section 2.5), to innate immune activation in infected and bystander cells. Whether abortive infections preferentially trigger antiviral responses remains an open question. However, maximal interferon induction by NS1-deficient influenza has been reported to require viral genome replication, and to be enhanced by nuclear export [91,92], suggesting that infections stalling before these steps may induce weaker rather than stronger responses, though this remains to be directly tested at the level of individual infection trajectories. Resolving whether abortive infections preferentially trigger antiviral responses will require approaches that can simultaneously track infection stage and antiviral response activation within the same cell over time.

Studies of other viruses have extended time-resolved analysis of infection and antiviral response activation to individual infection trajectories and multicycle spread. The use of recombinant vesicular stomatitis virus (VSV) expressing fluorescent reporters, together with cells carrying an IFIT2-promoter-driven fluorescent reporter, enabled live visualization of viral spread and antiviral response activation across infected cell layers [93]. These measurements showed that viral spread was slower when antiviral signaling remained intact during multicycle infection. A complementary live-cell study of encephalomyocarditis virus (EMCV) infection combined virus imaging with an IFIT1 reporter, allowing simultaneous monitoring of viral replication and antiviral response activation in individual cells [14]. Analysis of individual infection trajectories revealed that cells with early, low-level viral replication were the most likely to activate antiviral signaling. The combined single-cell observations of EMCV infection and antiviral responses support a model in which antiviral signaling is favored when early viral replication generates sufficient PAMPs before viral immune-evasion mechanisms become fully established. This mirrors the situation in IAV, where antiviral activation is likewise associated with replication proceeding without effective antagonism, but where the timing of activation relative to the establishment of NS1-mediated evasion remains poorly resolved at the single-cell level. Together, these studies show how single-cell imaging can be used to resolve the temporal relationship between viral replication, antiviral signaling, viral immune evasion, and the resulting effect on virus spread.

### 4.2. Imaging Spatial Mechanisms of Antiviral Response Activation

Bulk measurements of antiviral signaling components do not reveal where viral PAMPs, host sensors, and signaling molecules assemble during innate immune activation. Imaging preserves this spatial context, allowing the localization of viral and host signaling components to be related to signaling outcomes [94,95]. NS1 is the principal IAV innate immune antagonist and interferes with several components of antiviral signaling [81,96]. Consistently, cells infected with NS1-deficient virus are more likely, but not guaranteed, to activate immune responses [6,92]. IF microscopy revealed that NS1 suppresses IFN expression by inhibiting nuclear accumulation of interferon regulatory factor 3 (IRF3), directly linking altered protein localization to impaired antiviral signaling [97]. Complementary work showed that cytosolic NS1 binds the E3 ubiquitin ligase TRIM25 to inhibit the RIG-I ubiquitination required for effective signaling to IRF3 [98]. A non-peer-reviewed preprint used STED super-resolution imaging to examine the spatial organization of TRIM25 in NS1-containing cytosolic aggregates, suggesting a spatial mechanism by which NS1 disrupts RIG-I signaling [99]. IF imaging of antiviral stress granule (avSG) markers also showed that NS1 inhibits the formation of avSGs [100,101], dynamic RNA-protein assemblies that concentrate antiviral signaling molecules such as TRIM25, RIG-I, and PKR. Together, visualization of IRF3 nuclear accumulation, TRIM25 sequestration in NS1-containing aggregates, and antiviral stress-granule formation reveals how NS1 disrupts the localization and assembly of antiviral signaling components.

### 4.3. Imaging Stage-Specific Mechanisms of Antiviral Restriction

Antiviral restriction factors impair productive infection, but the stage of the viral life cycle at which restriction occurs and the underlying molecular mechanism are often unclear. Imaging can localize these stage-specific effects, providing insight into how ISGs suppress virus replication. This is well illustrated by the interferon-induced transmembrane protein IFITM3, which restricts IAV infection [89]. Single-particle live-cell imaging of fluorescently labeled virions was used to determine which stage of entry is targeted by IFITM3. IFITM3 overexpression did not prevent lipid mixing between viral and endosomal membranes but blocked release of viral contents into the cytosol. Distinguishing lipid mixing from cytosolic content release localized the IFITM3-mediated block between hemifusion and fusion pore formation [102]. A second important interferon-stimulated restriction factor in IAV infection is human MxA, which imposes a particularly strong barrier to many avian IAVs. Several studies connect MxA activity to the viral NP protein [103,104], but place MxA-mediated restriction at different stages of infection. Imaging implicated MxA in blocking nuclear accumulation of incoming vRNPs [105]. More recent work further implicated human MxA in the cytoplasmic sequestration of newly synthesized vRNPs, preventing their trafficking to the plasma membrane [106]. The reported effects at entry and during late-stage vRNP trafficking indicate that MxA may act at multiple stages of infection. It remains unclear whether both blocks operate within the same infected cell, or whether the dominant restriction point depends on virus strain, host-cell state, or experimental setup. More broadly, imaging can identify the stage of the viral life cycle perturbed by antiviral restriction by comparing the localization and trafficking of viral components in restricted and unrestricted infections.

## 5. Concluding Remarks

Throughout this review, we describe imaging not as a single methodology, but as a collection of complementary approaches that reveal distinct aspects of virus infection (Table 2). Fluorescence microscopy resolves the spatial organization of viral and host components, live-cell imaging establishes the temporal order of infection events, RNA imaging indirectly measures viral transcription and genome replication, and tissue-scale imaging relates intracellular infection to changes in tissue organization and function. Together, these approaches extend population-averaged measurements by enabling direct observation of individual infection trajectories and relationships between viral and host processes that would otherwise remain obscured. Imaging provides direct evidence for the measured observable, but not necessarily for the biological mechanisms underlying those observations. For example, colocalization does not establish molecular interaction, morphology does not define function, and RNA localization does not imply translation. Likewise, fluorescence measurements alone cannot resolve membrane topology or molecular mechanism. Interpreting imaging data therefore requires matching biological conclusions to the observable being measured and supporting mechanistic models with independent functional evidence. The Rab11-vRNP inclusions discussed in Section 3.4 illustrate this principle well: live imaging readouts consistent with liquid-like behavior are equally compatible with alternative clustering mechanisms, and thus orthogonal perturbation experiments are required to determine whether these inclusions represent genuine liquid condensates.

Because different imaging approaches measure complementary observables, integrating these measurements can provide a more complete picture than any single approach alone. Measuring complementary parameters is particularly informative because viral and host-cell processes are themselves highly interconnected. Virus infection can remodel host cells, while host-cell remodeling and antiviral responses in turn influence the progression and outcome of infection. Yet specialized imaging approaches often examine viral replication, host-cell remodeling, antiviral signaling, and tissue organization separately. Combining measurements of these processes within the same cells or tissues would allow their spatial and temporal relationships to be examined directly. Such integrated measurements could link early intracellular events to subsequent productive or nonproductive outcomes, identify candidate causal relationships that can be evaluated using complementary evidence, and provide a more integrated understanding of how viral and host processes together shape infection outcome.

## Figures and Tables

**Figure 1:**
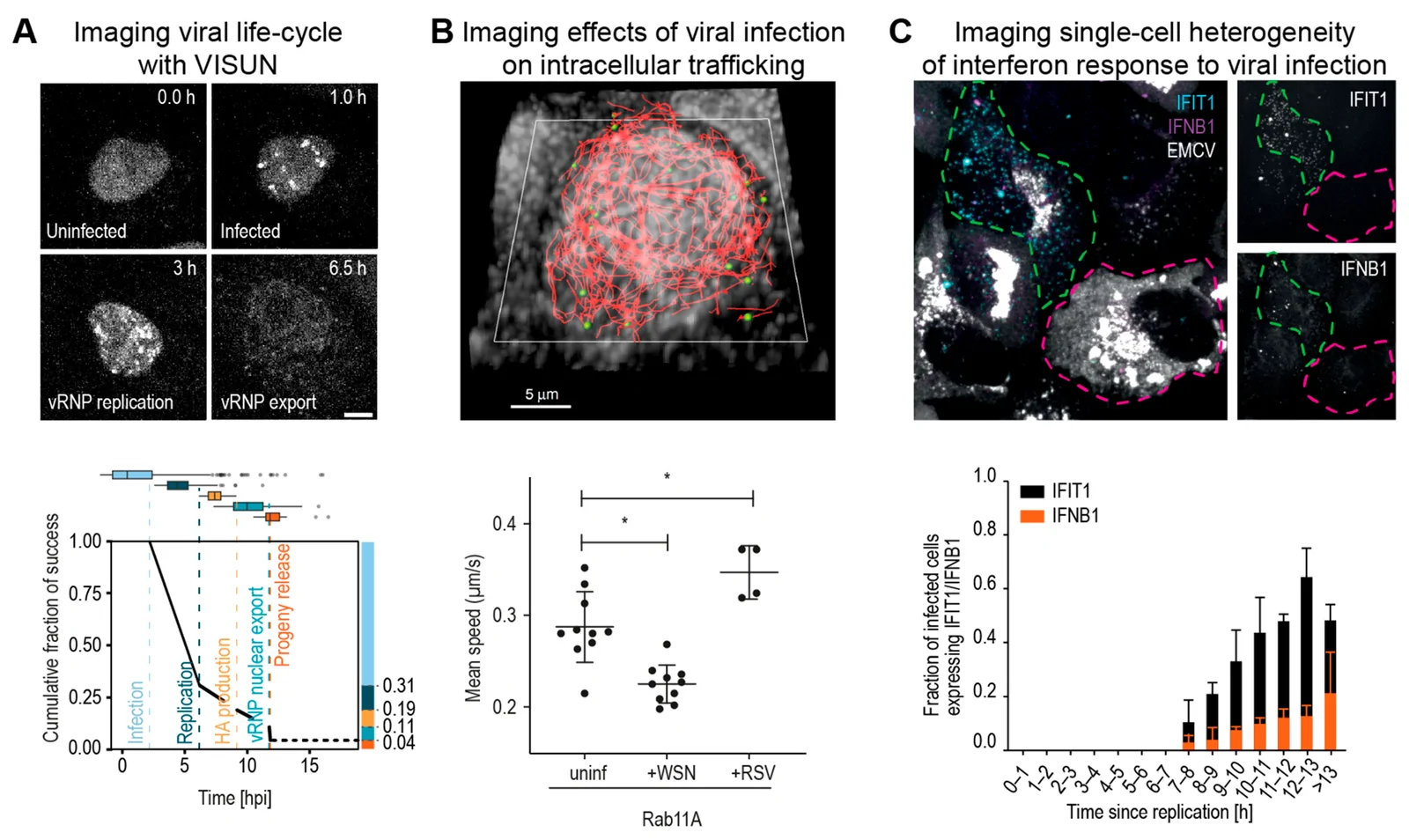
Examples of imaging techniques related to IAV lifecycle tracking, host cell remodeling, and antiviral responses. (**A**) VISUN follows successive stages of the IAV life cycle in single cells, including vRNP replication and nuclear export (top), and reveals heterogeneity in the timing and completion of infection stages (bottom). (**B**) Volumetric live-cell imaging and single particle tracking reveal virus-specific changes in Rab11A transport (top), illustrated by reduced Rab11A speed during IAV infection (WSN) and increased speed during respiratory syncytial virus infection (RSV) (bottom). Mean ± standard deviation (SD) of each group is shown (* implies *p* < 0.05, comparisons performed using one-way ANOVA with Turkey’s multiple test). (**C**) Combined imaging of viral replication and antiviral gene expression (top) reveals cell-to-cell heterogeneity in IFIT1 and IFNB1 induction over the course of infection (bottom). Green outline highlights an infected cell displaying IFIT1 and IFNB1 induction. Red outline highlights an infected cell which fails to display IFIT1 or IFNB1 induction. Panel (**A**) adapted from Rabouw et al. (2026) [12] with permission from Elsevier; panel (**B**) adapted from Bhagwat et al. (2020) [13]; panel C adapted from Bruurs et al. (2023) [14]. Panels (**B**,**C**) are adapted under the Creative Commons Attribution 4.0 International License (http://creativecommons.org/licenses/by/4.0/ accessed on 24 September 2026).

**Figure 2:**
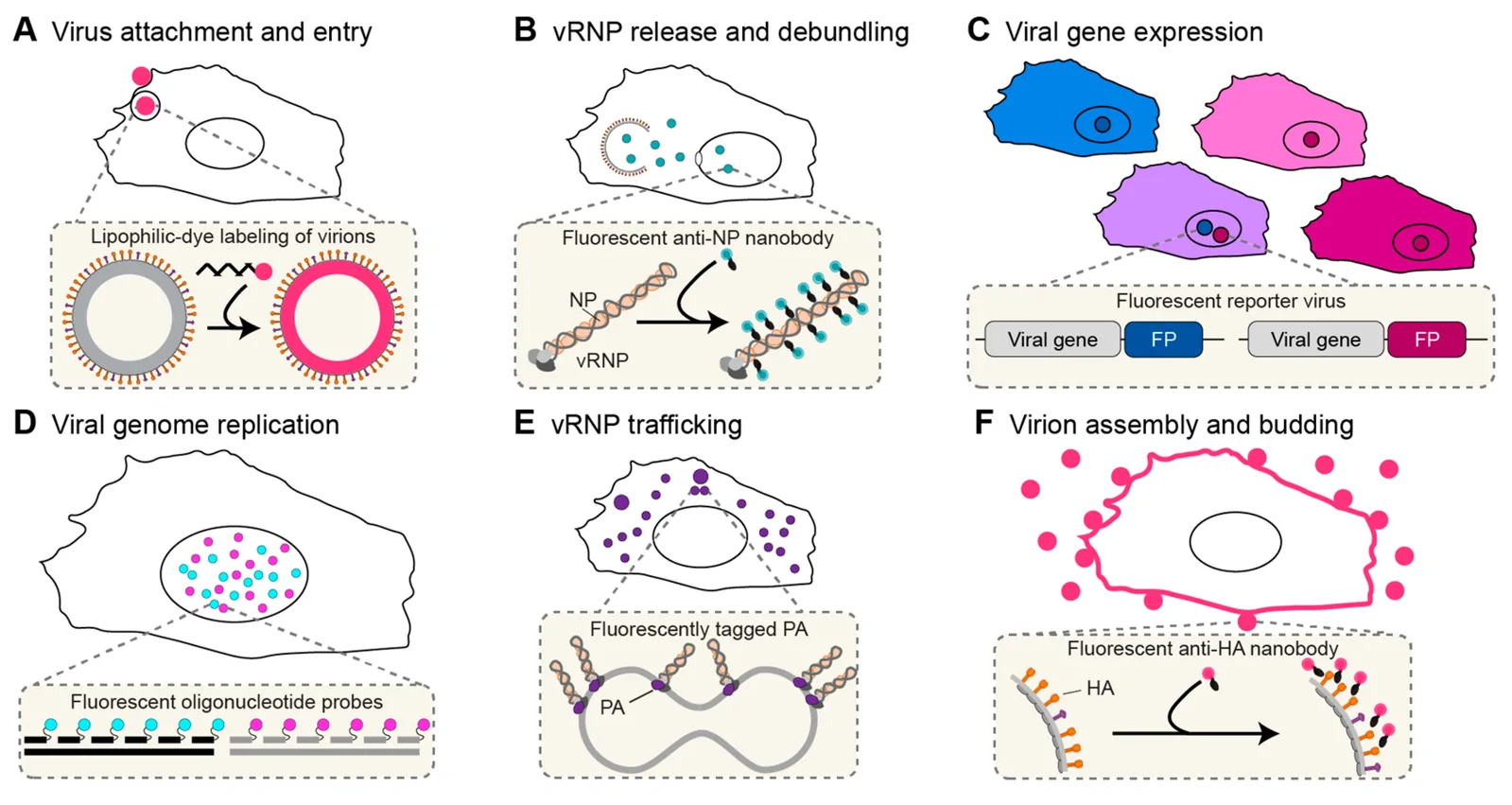
Selected imaging strategies for visualizing the influenza A virus life cycle. (**A**) Lipophilic membrane dyes label virions for single-particle tracking during attachment and entry. (**B**) Fluorescent anti-NP nanobodies, expressed in host cells, visualize incoming vRNPs and their release and debundling after membrane fusion. (**C**) Fluorescent reporter viruses encode fluorescent proteins as indirect, time-resolved readouts of viral gene expression and can distinguish singly infected and coinfected cells. (**D**) Fluorescent oligonucleotide probes detect viral nucleic acids; here, they illustrate spatial and quantitative analysis of viral genome replication. (**E**) Fluorescent tagging of PA enables live tracking of vRNP trafficking; the inset illustrates fusion between two PA-labeled, vRNP-containing structures. (**F**) Extracellular fluorescent anti-HA nanobodies label HA exposed on budding progeny virions, providing a live readout of virion assembly and release. Dashed lines connect each infection stage to an enlarged schematic of an exemplary imaging strategy.

**Table 1:** Imaging approaches for studying virus infection: practical requirements and constraints.

Imaging Approach	Labeling Strategy	Specimen and Model Requirements	Applicable to Living Cells	Temporal Constraints	Key Limitations	Typical Imaging Scale *
Exogenous labeling of virions or viral components	Labeling by dyes, quantum dots, or binding probes	Targets accessible during labeling; no genetic engineering required	Yes	No maturation delay; timing depends on probe and acquisition kinetics	Bleaching and phototoxicity; labels may perturb the target’s localization or function; incomplete labeling may lead to underestimation of target abundance or detection bias	Small to medium
Intracellular live labeling of viral components	Host cell expression of fluorescent binders targeting viral proteins	Host cells amenable to genetic engineering	Yes	Binding kinetics may delay readout	Bleaching and phototoxicity; binders may perturb the target or cell	Small to medium
Fixed-cell target-specific imaging	Labeling by target-specific antibodies or sequence-specific probes	Fixed, permeabilized cells or tissues; no genetic engineering required	No	Snapshot only; dynamics inferred from separate samples	Fixation and preparation artifacts; incomplete labeling may lead to underestimation of target abundance or detection bias	Small to large
Genetically encoded viral reporters	Expression of a virally encoded fluorescent or luminescent reporter	Engineered virus; validation of viral fitness and genetic stability	Yes	Reporter maturation and persistence limit temporal accuracy	Bleaching and phototoxicity; reporter insertion may alter viral fitness or gene expression	Small to large
Genetically encoded fluorescently tagged viral proteins	Genetic fusion of a fluorophore to a viral protein of interest (POI)	Engineered virus; validation of viral fitness and genetic stability	Yes	Reporter maturation and persistence limit temporal accuracy	Bleaching and phototoxicity; reporter insertion may alter viral fitness, gene expression, or localization of viral POI	Small to large
Functional reporters and tracer assays	Host cell expression of reporters or labeling through exogenous tracers	Host models compatible with reporter expression or tracer delivery	Yes	Assay-dependent, from seconds to hours	Bleaching and phototoxicity; reporters or tracers may perturb the measured process	Small to large; assay-dependent

* Small, medium, and large refer approximately to imaging of single cells, multicellular fields or cell layers, and cell populations or tissues, respectively. Ranges describe typical implementations rather than absolute limits. Notes. Signal-to-noise, imaging depth, and field of view depend primarily on the imaging modality rather than the labeling strategy, and are therefore not tabulated here.

**Table 2:** Imaging approaches for studying virus infection: biological readouts and interpretive limitations.

Imaging Approach	Fixed	Live	Direct Readout	Viral Infection	Host Remodeling or Function	Antiviral Responses	Key Interpretive Limitation
Single-particle virion imaging	○	●	Virion position, movement, membrane fusion, and content release	Attachment, entry, trafficking, and fusion	—	Entry restriction	Detected virions are not necessarily infectious
Protein imaging (IF, super-resolution)	●	—	Selected protein abundance and localization	Viral-protein abundance and localization	Protein and organelle redistribution	Localization of antiviral signaling factors	Does not establish interaction or function
RNA imaging (smFISH, multiplexed)	●	—	RNA abundance, identity, and localization	Viral transcription, genome replication, RNA localization, and genome assembly	Host RNA abundance and localization	Transcription of antiviral genes	Does not directly measure RNA translation, integrity, or function
Genetically encoded viral reporters	○	●	Tagged-protein localization or reporter expression	Viral gene expression, protein localization, coinfection, and spread	—	—	Reporter expression may not fully reflect native viral gene expression
Nanobody-based imaging	○	●	Fluorescently labeled vRNPs or surface-accessible viral proteins	vRNP trafficking, genome replication, nuclear export, and budding	—	—	Nanobody binding may alter the localization or function of the target protein (e.g., anti-HA nanobodies neutralize virions [107])
Functional reporters and biosensors	○	●	Promoter activity, translational activity, membrane potential, or biochemical activity	Viral replication and gene expression	Organelle function and host translation	Antiviral response activation and signaling dynamics	Reporter activity may not fully reflect endogenous biological activity
Cargo and tracer assays	○	●	Cargo transport and pathway function	—	Cargo transport and pathway activity	—	Artificial cargo may not faithfully mimic native viral components
Tissue-scale imaging	●	●	Three-dimensional organization and longitudinal dynamics across multicellular samples	Viral dissemination and spread	Tissue architecture, composition, and function	Intercellular antiviral signaling	Limited molecular and ultrastructural detail
Ultrastructural and correlative imaging	●	—	Membrane topology, structural organization, and molecular–structural correspondence	Replication-compartment architecture, virion structure, and budding	Membrane and organelle remodeling	—	Ultrastructure alone does not reveal molecular composition or function

Markers: ● primary application; ○ possible or secondary application; — no direct application discussed in this review. Abbreviations: smFISH, single-molecule fluorescence in situ hybridization; vRNP, viral ribonucleoprotein. Note: The categories reflect how imaging approaches are applied in practice and therefore span imaging modalities, labeling strategies, and sample scales. They are overlapping rather than mutually exclusive, and a single experiment may combine several.

## Data Availability

No new data were created or analyzed in this study. Data sharing is not applicable to this article.
